# High-temporal resolution metabolic connectivity resolved by component-based noise correction

**DOI:** 10.1177/0271678X261431043

**Published:** 2026-03-13

**Authors:** Murray B Reed, Samantha Graf, Matej Murgaš, Benjamin Eggerstorfer, Christian Milz, Leo R Silberbauer, Pia Falb, Elisa Briem, Alexandra Mayerweg, Gabriel Schlosser, Sebastian Klug, Lukas Nics, Godber M Godbersen, Sazan Rasul, Marcus Hacker, Andreas Hahn, Rupert Lanzenberger

**Affiliations:** 1 Department of Psychiatry and Psychotherapy, Medical University of Vienna, Vienna, Austria; 2 Comprehensive Center for Clinical Neurosciences and Mental Health (C3NMH), Medical University of Vienna, Vienna, Austria; 3 Division of Nuclear Medicine, Department of Biomedical Imaging and Image-Guided Therapy, Medical University of Vienna, Vienna, Austria

**Keywords:** Molecular connectivity, band-pass, [^18^F]fludeoxyglucose, [^18^F]FDG, within-subject connectivity

## Abstract

Recent advances in functional PET (fPET) enable modeling of metabolic processes with second-level temporal resolution, opening applications such as imaging molecular connectivity comparable to fMRI. However, high-temporal fPET is more noise-sensitive, making meaningful signal extraction challenging. We developed a component-based preprocessing method adapted from fMRI, which models structured noise with tissue-specific regressors and removes low-frequency uptake trends (CompCor). This approach was applied to 20 high-temporal [^18^F]FDG–fPET scans from a long-axial PET/CT system (1 s frames) and 16 scans from a PET/MR scanner (3 s frames). Filtering methods were compared across frequency bands, and their effects on metabolic connectivity (M-MC) assessed. Connectivity was strongly influenced by filter strategy and scanner type. CompCor produced more consistent, structured networks than standard bandpass filters. Intermediate frequency bands (0.01–0.1 Hz) gave the most reliable connectivity across PET/CT and PET/MR (*r* = 0.89), while high-sensitivity PET/CT also revealed structured patterns at 0.1–0.2 Hz. Compared to fMRI, fPET networks appeared more spatially cohesive but less differentiated. In sum, high-temporal [^18^F]FDG–fPET enables high within-scan reliability estimation of resting-state M-MC when paired with appropriate denoising, opening a new avenue in molecular imaging. Scanner characteristics and preprocessing critically affect signal quality, while our physiologically informed pipeline improves comparability across systems and studies.

## Introduction

Understanding how brain regions interact remains a core question in neuroscience. Functional connectivity, measured with blood oxygenation level dependent (BOLD) functional magnetic resonance imaging (fMRI), has offered one way to study these interactions by capturing the temporal correlation of hemodynamic signals.^1,2^ However, the BOLD signal is an indirect measure of neuronal activity.^3^ It reflects a combination of changes in blood flow, blood volume, and oxygen consumption rather than the specific metabolic demands that sustain neural function.^4–6^

In contrast, [^18^F]fluorodeoxyglucose–positron emission tomography ([^18^F]FDG–PET) provides a more direct measure of glucose metabolism.^7^ FDG–PET has traditionally been used to create static maps of regional glucose uptake,^8,9^ offering specific insight into brain function. While clinically useful, static PET lacks the temporal resolution to capture moment-to-moment changes that define dynamic brain function^10^ thus may have different predictive value as compared to functional PET (fPET) data.^11,12^ Recent advances have started to shift this limitation using fPET.^13,14^ The adoption of bolus plus constant infusion protocols, along with improved image reconstruction and processing, has increased the temporal resolution of fPET from minutes to seconds.^15–18^ With appropriate acquisition and reconstruction, metabolic signals can now be sampled in high-temporal frames, making it possible to detect acute task-evoked changes in metabolic activity.^19^

Beyond task-based responses, this framework has sparked interest in studying resting-state metabolic connectivity (M-MC).^11,20–23^ As resting-state fMRI reveals functional networks in absence of external stimulation, high-temporal resolution fPET offers a promising approach to characterize the metabolic network counterpart. This in turn could provide a molecular-level view of brain network structure in healthy individuals but also in clinical populations, complementing the hemodynamic signal and capturing aspects of neural function not seen in the fMRI signal.^24^ Previous studies have demonstrated the feasibility of estimating metabolic connectivity using [^18^F]FDG–fPET with a temporal resolution of 16 s.^11^ However, insights from resting-state fMRI suggest that spontaneous neuronal fluctuations contributing to functional connectivity predominantly occur at higher frequencies. This raises the question of whether increasing the temporal resolution of fPET could improve the characterization of dynamic metabolic network interactions. However, such a shift toward higher temporal resolution also introduces methodological challenges, including reduced signal-to-noise ratio and increased sensitivity to preprocessing choices. Yet the shift to high temporal resolution fPEt also introduces analytical challenges.^25^ Short image frames in the range of seconds substantially reduce detected counts, thereby lowering the signal-to-noise ratio and increasing vulnerability to motion, physiological artifacts, and scanner variability.^25,26^ Crucially, resting-state analyses rely on data-driven methods that are more sensitive to artifacts and the corresponding preprocessing choices.^27–31^ Thus, the ability to detect reliable connectivity patterns at rest depends heavily on how the data is filtered and denoised.

Existing PET preprocessing pipelines have been developed for lower temporal resolution.^32^ While still effective in that setting, they may not preserve the temporal structure needed for connectivity analyses at higher temporal resolution. In this case, filtering becomes a trade-off between reducing noise and preserving the signal. Too much filtering may remove biologically relevant fluctuations. Too little, and noise may falsely contribute to connectivity signals.^33,34^ The problem is further complicated by limited knowledge of which frequency bands are most relevant for metabolic signals.

The present study aims to address key analytical limitations in high-temporal-resolution [^18^F]FDG–fPET by: (i) introducing an integrated filtering framework for robust assessment of moment-to-moment fluctuations; (ii) computing metabolic connectivity at a previously unprecedented temporal resolution of 1 s; (iii) evaluating the contribution of distinct frequency bands to metabolic connectivity; and (iv) assessing generalizability by comparing results with data from a conventional PET/MR system. These advancements are enabled by applying the proposed method to data acquired on a next-generation large axial field-of-view (LAFOV) PET/CT scanner operating in ultra-high-sensitivity mode. The integrated filtering approach, adapted from the fMRI domain, consolidates multiple preprocessing steps, including temporal detrending, regression of motion and physiological noise, and frequency-specific band separation, into a unified temporal filter. Together, our work aims to provide a robust and widely applicable approach to assess metabolic connectivity in the human brain.

## Materials and methods

### Participants

Twenty healthy participants (mean age ± SD = 24.8 ± 3.6 years; 11 female) underwent a single [^18^F]FDG–PET/CT scan using a ultra-high sensitivity LAFOV scanner (Siemens Vision Quadra), followed by an fMRI scan on a Siemens MAGNETOM Prisma 3 T. An additional 16 healthy volunteers (mean age ± SD = 26.9 ± 8.2 years; seven female) completed two [^18^F]FDG scans on a 3 T PET/MR system (Siemens Biograph mMR; Siemens Healthineers, Germany).

General health was evaluated through a structured medical assessment. This included medical history, physical examination, electrocardiogram, and routine laboratory testing. Psychiatric screening was conducted using the Structured Clinical Interview for DSM-IV Axis I Disorders (SCID-I) to rule out current or past psychiatric diagnoses.

Exclusion criteria included any current or previous severe medical condition, psychiatric disorder, psychopharmacological treatment (past 6 months), or contraindications to PET/MRI. These included metallic implants, claustrophobia, and prior radiation exposure. Urine drug tests were performed at screening. For female participants, urine pregnancy tests were conducted at screening and again before each scan.

All participants provided written informed consent and received financial compensation for their time. Both studies were approved by the ethics committee of the Medical University of Vienna (EK 1307/2014 and 1642/2022) and conducted in accordance with the Declaration of Helsinki. The investigation forms part of a larger study registered prior to participant recruitment at clinicaltrials.gov (NCT02711215 and NCT06243783, respectively).

### Study design

For the LAFOV PET/CT study, participants were positioned with the head centered in the scanner bore to optimize count sensitivity. Data was acquired in ultra-high sensitivity mode (NEMA phantom: ~176 kcps/MBq^35,36^). Each session began with a topogram and a low-dose CT scan, followed by a 25-min resting-state PET acquisition.^37^ During the scan, participants were instructed to remain awake with their eyes open, maintain fixation on a black crosshair presented on a gray background, and allow their thoughts to wander freely without focusing on any specific task. Radiotracer administration followed a bolus plus constant infusion protocol, initiated simultaneously with the start of PET acquisition (see data acquisition).

In the separate PET/MR cohort, data from a single scan were used as part of a randomized, double-blind, cross-over design involving administration of a placebo on a Siemens Biograph mMR 3 T (NEMA phantom: ~15.0 kcps/MBq^38^). Each session began with a high-resolution T1-weighted structural MRI scan. This was followed by a 50-min simultaneous PET and BOLD–fMRI acquisition. The radiotracer protocol was identical to the PET/CT study, with bolus and infusion starting at the beginning of the scan. At 20 min, the pharmacological agent was administered intravenously. Throughout the acquisition, participants maintained visual fixation and were instructed to remain still and let their thoughts wander. For the present analysis, only the first 20 min of the fPET data were included. This matched the acquisition window of the PET/CT study and ensured that pharmacological effects did not confound the results. To avoid contamination of connectivity estimates by the rapid global tracer uptake following bolus injection, only time points acquired after 10 min post-infusion were included in all metabolic connectivity analyses. This time window excludes the initial non-stationary uptake phase and (together with the filtering methods) minimizes spurious correlations driven by shared tracer delivery rather than dynamic metabolic fluctuations.

### Data acquisition and reconstruction

[^18^F]FDG was synthesized on each study day at the Department of Biomedical Imaging and Image-guided Therapy, Division of Nuclear Medicine, Medical University of Vienna.

At the start of fPET acquisition, [^18^F]FDG was injected through a cubital vein as a 1-min bolus, followed by constant infusion for the rest of the scan using a shielded pump (Syramed mSP6000; Arcomed, Switzerland). The dose was 5.1 MBq per kg body weight. Bolus speed was 816 ml/h. Infusion speed was 91.0 ml/h for the LAFOV system (total infusion time: 25 min) and 29.5 ml/h for the PET/MR system (total infusion time: 50 min), each with a bolus and infusion ratio of 20:80.^16^ All fPET data on the PET/MR system were acquired in list mode, allowing frame lengths to be defined during reconstruction.

The T1-weighted structural image acquired utilized a magnetization-prepared rapid gradient echo (MPRAGE) sequence (TE 4.21 ms, TR 2200 ms, voxel size 1 × 1 × 1.1 mm, matrix 240 × 256, 160 slices, flip angle 9°, TI 900 ms, duration 7.72 min).

In the LAFOV PET/CT protocol, a low-dose CT was acquired after breath-hold instructions (120 kVp, 20 mA, CareDose4D and CarekV enabled). CT images were reconstructed with 0.98 × 0.98 × 4 mm voxels. Thereafter, fPET data was acquired in list mode. MRI data for the LAFOV study was recorded on a Siemens Prisma 3 T scanner with a 64-channel head coil. Functional MRI images for the LAFOV study were acquired with an EPI sequence (TE 30 ms, TR 900 ms, field of view 200 × 200 mm, resolution 80 × 80 pixels, 48 axial slices of 2.4 mm thickness, multiband factor 4, TA: 20:07 min).

All fPET data were reconstructed using Siemens’ E7 Tools. LAFOV fPET data was reconstructed using 3D-TOF OP-OSEM with four iterations and five subsets into 1500 frames of 1 s. CT-based attenuation correction was applied. PET/MR data was reconstructed with OP-OSEM using three iterations and 21 subsets into 400 frames of 3 s each. Here, attenuation correction was based on a pseudo-CT derived from the T1 image.^39^ Fully quantified CMRGlu estimates were not derived, as the focus of the present work was on high-temporal-resolution analysis of relative FDG signal fluctuations required for metabolic connectivity analyses, rather than on absolute metabolic rate quantification.

For the comparison between LAFOV PET/CT and PET/MR data, the LAFOV data (1 s frames) was downsampled after reconstruction to match the 3 s frame duration of the PET/MR acquisitions.

### CompCor filter

**Figure 1.**
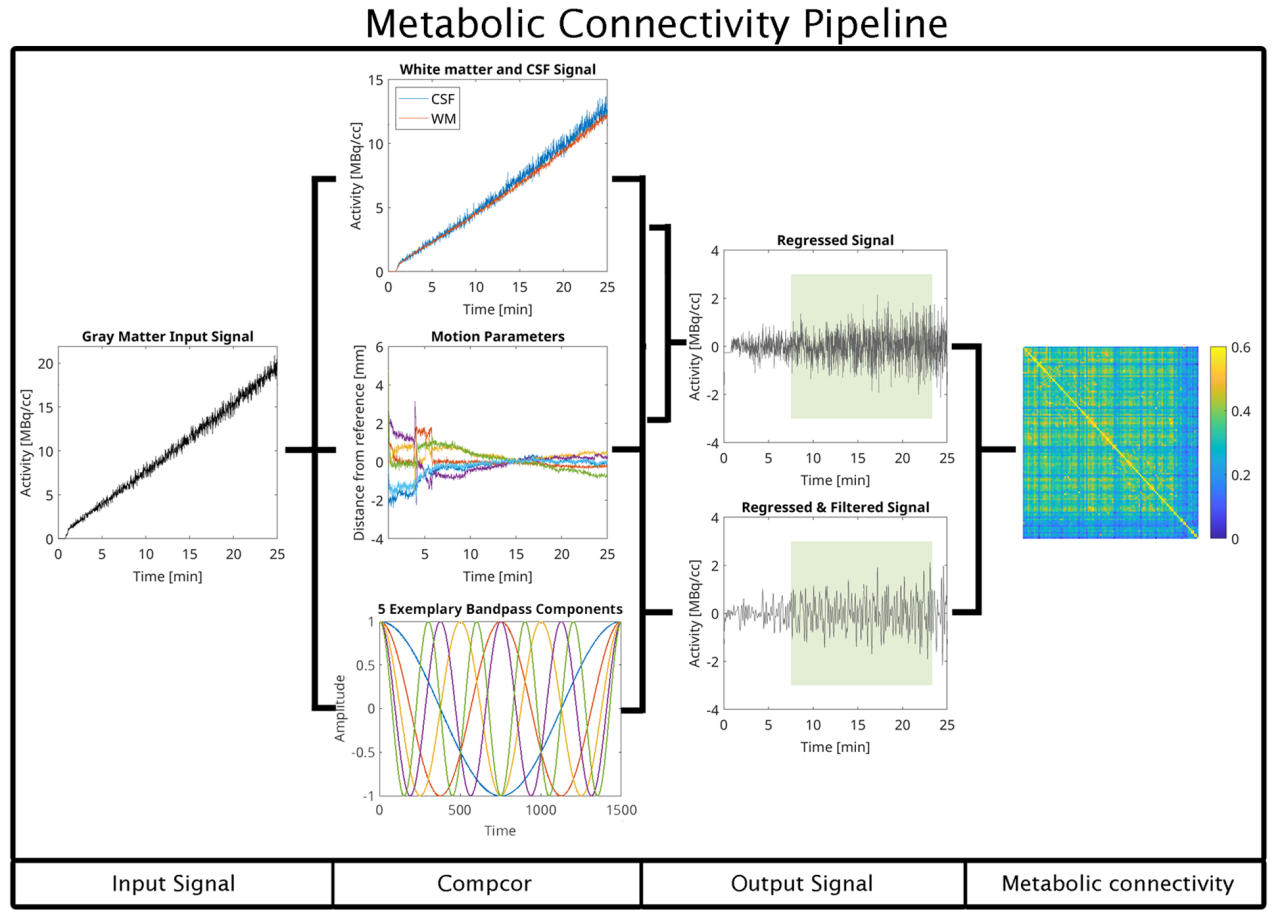
Computation of metabolic connectivity using the CompCor-based preprocessing pipeline. The approach operates in a voxel-wise fashion to prepare high-temporal resolution fPET data for connectivity analysis. After standard preprocessing, the gray matter signal is isolated and submitted to the CompCor algorithm alongside head motion parameters and, optionally, a specified frequency band of interest. The algorithm first extracts nuisance signals from anatomically defined white matter and cerebrospinal fluid masks. It then derives a set of principal components that capture the majority of variance within these non-neuronal compartments, reflecting physiological and scanner-related noise. To improve correction for head motion, the six rigid-body motion parameters are expanded by computing their temporal derivatives, squared terms, and squared derivatives, again followed by principal component analysis. This extended motion model allows for the capture of both linear and nonlinear movement-related artifacts. If a bandpass filter is defined, the corresponding sine and cosine basis functions are generated to model frequencies outside the desired spectral window. All nuisance regressors, including white matter and CSF components, expanded motion parameters, and optional frequency regressors, are entered into a single multiple regression model. This simultaneous regression removes confounding sources of variance in a unified step, minimizing the risk of reintroducing noise through sequential filtering. The resulting time series are thus cleaned of motion artifacts, physiological noise, non-neuronal fluctuations, and undesired frequency components. These denoised signals provide a robust basis for the estimation of metabolic connectivity metrics. Representative subject-level [^18^F]FDG time series shown in this figure illustrate the raw time-activity curve (input signal), extraction of the white matter removal of the cumulative tracer uptake component and structured nuisance variance (CompCor), resulting in stationary signals suitable for metabolic connectivity estimation (output signal).

To remove structured noise from the fPET data and improve signal quality, we implemented a variant of the data-driven CompCor (component-based noise correction) method, originally developed for fMRI,^28^ and adapted it for use with fPET data. This approach is designed to reduce physiological and motion-related noise by identifying and removing components of the signal that are unlikely to originate from neural activity. Specifically, fluctuations associated with white matter, cerebrospinal fluid (CSF), and head motion were treated as nuisance regressors. Although rigid-body realignment reduces gross head motion, residual motion-related variability remains in high-temporal-resolution functional imaging data like fPET and fMRI. To account for this, the Friston 24-parameter model, which includes the six rigid-body motion parameters, their temporal derivatives, and the corresponding squared terms, was included as a nuisance regressor within the CompCor framework, capturing variance not eliminated by spatial realignment and improving correction of motion-induced signal fluctuations.^40^ Tissue-specific nuisance signals from white matter and CSF were extracted from SPM’s tissue probability maps. The masks were thresholded to retain only the upper 5% of voxels with the highest respective tissue probability, ensuring exclusion of gray matter regions and minimize the inclusion of signal from tissue of interest. Principal component analysis was then used to capture the dominant patterns of non-neural variability, which were regressed out of the fPET data (see below). This also removes the cumulative tracer uptake from the signal to allow for accurate M-MC estimation.^10^ This method enhances the sensitivity of molecular connectivity (MC) analyses by reducing structured noise and isolating signal changes more closely associated with underlying neural processes. See Figure 1 for a graphical overview of the CompCor method.

Briefly, the CompCor approach removes structured nuisance variance from the dynamic [^18^F]FDG signal by regressing out principal components derived from regions dominated by non-neuronal contributions, such as CSF and white matter. These components capture global tracer delivery effects, residual kinetic trends, and physiological noise that are shared across brain regions but are not directly related to local metabolic interactions. By removing these components from each voxel or region-wise time series, CompCor yields signals that are more suitable for correlation-based metabolic connectivity analyses.

### Preprocessing of fPET data

Preprocessing and quantification of all fPET data followed procedures established in previous studies.^16,41^ Head motion correction was performed using SPM12 (https://www.fil.ion.ucl.ac.uk/spm) with the quality setting at “best” Images were realigned to a motion-free reference created from a stable time segment late in the scan, defined as frames later than 15 min. The mean PET image was coregistered to each participant’s structural MRI, which in turn was spatially normalized to MNI space. These transformations were then applied to the full dynamic fPET series. Normalized data were either smoothed with an 8 mm FWHM Gaussian kernel or filtered with an extended dynamic non-local means (edNLM) filter (for PET/MR data only, due to the lower scanner sensitivity) as the latter proved most efficient for task-based fPET analyses.^42^ The edNLM filter used kernels of 3 × 3 × 3 voxels × three frames and 3 × 3 × 3 voxels × five frames, followed by 5 mm Gaussian smoothing.^42^ Spatial normalization was applied prior to CompCor and regional time-series extraction to enable anatomically consistent definition of gray matter, white matter, and cerebrospinal fluid masks across subjects. As CompCor operates on relative temporal fluctuations, these signals are preserved under spatial normalization, while performing nuisance regression and parcellation in common space avoids additional inverse transformations and unnecessary interpolation.

After preprocessing, the time series from each participant was filtered voxel-wise using the CompCor method, or region-wise using a fourth-order Butterworth band-pass filter. The Butterworth filter was implemented as an infinite impulse response filter in MATLAB and included correction for motion parameters through partial correlation. The data were filtered into the following frequency bands (see results): 0.001–0.01, 0.01–0.1, and 0.1–0.2 Hz for the LAFOV dataset, and 0.001–0.01, 0.01–0.1, and 0.1–0.16 Hz for the PET/MR dataset. Band limits for the PET/MR data were adjusted to remain within the Nyquist frequency. These bands were selected by examining the power spectral density of each scan to identify the frequency ranges where signal peaks were observed. For analyses conducted within specific frequency bands, filter cutoff frequencies were adjusted accordingly to match the respective band limits.

Metabolic connectivity matrices were then calculated from the processed time series. For each participant, regional mean signals were extracted using a combined parcellation of the Schaefer 100 cortical parcels^43^ and 14 subcortical regions from the Harvard–Oxford atlas.^44^ Pearson correlation was used to compute the M-MC matrix for each subject filtered by CompCor. For Butterworth-filtered data, partial correlation was applied to account for motion parameters, which is inherently processed in the CompCor.

### Preprocessing of fMRI data

Physiological noise in the fMRI data (acquired at Siemens Prisma scanner) was reduced using PESTICA.^45^ Further preprocessing was performed in SPM12. Slice-timing correction was applied relative to the temporally middle slice. Images were then realigned to the mean image.

Spatial normalization used the standard MNI template. Images were resliced to 2.5 mm isotropic resolution, preserving approximate voxel volume. Nonlinear artifact reduction was carried out with the BrainWavelet Toolbox,^46^ using the “chsearch” parameter set to “harsh” and a threshold of 20. This was optimized for unsmoothed data acquired with multi-band acceleration, which typically has reduced signal-to-noise ratio. All images were masked using a gray-matter template. Smoothing was applied using a Gaussian kernel three times the resliced voxel size (8 mm).

Following preprocessing, CompCor filtering was applied in a voxel-wise manner, similar to fPET. Regional mean signals were extracted using the same atlas parcellation as for fPET (Schaefer 100 + Harvard–Oxford subcortical). Functional connectivity was estimated using Pearson correlation on the data after removing the initial 5 min of data points.

### Statistical analysis

Mean metabolic and functional connectivity matrices were computed across subjects for visualization purposes. To assess the similarity between filtering approaches, Spearman correlations were calculated between CompCor and Butterworth filtering, and between Gaussian smoothing and edge-preserving non-local means (edNLM) filtering. Additional comparisons were made between scanner types (PET/MR vs LAFOV) and across frequency bands.

To investigate macroscale network structure of fPET and fMRI data, connectivity matrices were subjected to hierarchical clustering. The clustering used Ward’s linkage method and Euclidean distance as the similarity metric and was selected because it minimizes within-cluster variance and yields compact, interpretable clusters when applied to continuous-valued similarity matrices.^47^ The quality of the resulting dendrograms was evaluated using the cophenetic correlation coefficient, which quantifies how faithfully the clustering preserves the pairwise distances between elements.

In addition, connectivity matrices were clustered using agglomerative clustering with eight predefined clusters. This choice was based on the Yeo 7-network cortical parcellation,^48^ with one additional cluster assigned to subcortical regions. The goal was to enable a more direct comparison between metabolic and functional network structures.

To further evaluate the robustness and reliability of the MC estimates derived from CompCor and band-pass filtering methods, a test–retest analysis was performed using random data splits. The data were randomly divided into two halves for each subject, and reliability was assessed through the Pearson correlation coefficient between the mean connectivity matrices derived from the first and second halves of the data. This analysis was repeated across 5000 permutations to generate a distribution of reliability scores.

## Results

**Figure 2.**
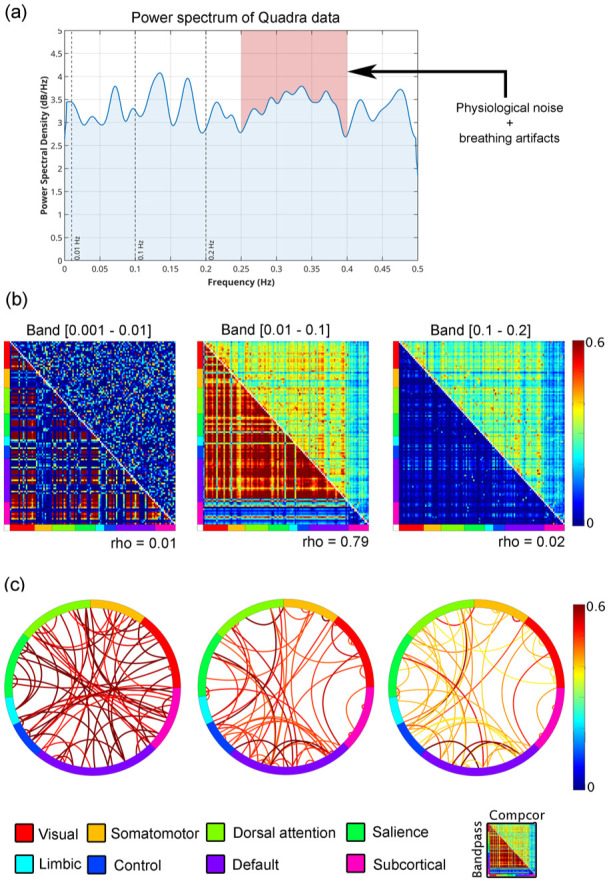
Overview of metabolic connectivity metrics estimated using CompCor and Butterworth band-pass filtering on high-temporal resolution fPET data acquired with a LAFOV PET/CT system: (a) shows an individual power spectral density estimates after removal of baseline uptake (blue). Standard frequency bands are depicted as dashed vertical lines for 0.01, 0.1, and 0.2 Hz. The red shaded area indicates the typical bands where physiological noise such as breathing artifacts occur,^58,59^ (b) displays the metabolic connectivity matrices, separated by frequency band. The lower triangle shows connectivity derived from the Butterworth filter. The upper triangle shows connectivity estimated using the CompCor method. Connectivity estimates derived using the Butterworth filter were overestimated in the lower frequency bands (0.001–0.01 and 0.01–0.1 Hz), and substantially underestimated in the higher frequency band (0.1–0.2 Hz). Notably, the 0.01–0.1 Hz band was the only range in which a strong linear correlation was observed between the two filtering methods, and (c) illustrates the strongest 5% of connections across each frequency band, based on CompCor-filtered data. Connections are grouped according to large-scale functional networks as defined by Thomas Yeo et al.,^48^ along with subcortical regions from the Harvard–Oxford atlas.^60^

**Figure 3.**
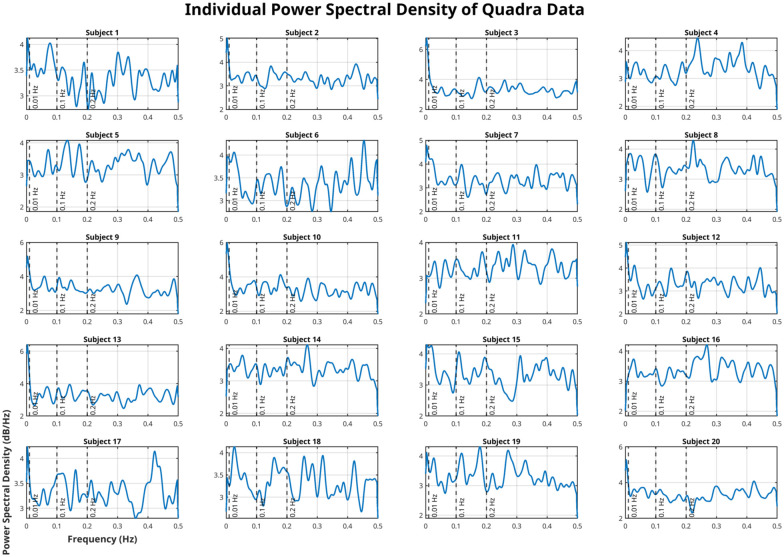
Overview of all particpants’ power spectra estimates after removal of baseline uptake. Standard frequency bands are depicted as dashed vertical lines for 0.01, 0.1, and 0.2 Hz.

High-temporal resolution metabolic fPET data acquired with the next-generation LAFOV PET/CT scanner exhibited distinct spectral features following baseline uptake correction. Power spectral density (PSD) analyses revealed individual and group-level peaks across multiple low-frequency bands (Figures 2(a) and 3). Inter-individual variability in the power spectra is expected in high-temporal-resolution fPET, as metabolic signals reflect a mixture of neuronal, physiological, and scanner-related fluctuations that do not conform to a uniform spectral profile across subjects.

Comparison of filtering methods revealed notable differences in estimated M-MC (Figure 2(b)). While the 0.01–0.1 Hz band yielded high similarity between the CompCor and Butterworth filter (Spearman’s rho = 0.79), both lower (0.001–0.01 Hz) and higher (0.1–0.2 Hz) bands showed no agreement (rho ⩽ 0.02). Butterworth-filtered data tended to overestimate connectivity in the lower two bands and underestimate it in the higher band relative to CompCor-filtered data. Visualization of the strongest 5% of CompCor-filtered connections highlighted distinct spatial organization across bands, consistent with known functional network architecture (Figure 2(c)). Notably, the 0.1–0.2 Hz band exhibited similarly structured resting-state-like connectivity patterns as compared to the 0.01–0.1 Hz band, and correlated highly (rho = 0.94) when using CompCor. For the Butterworth-filtered data, the correlation between these frequency bands was markedly lower (rho = 0.29). However, the connectivity strength was decreased as within and between network connectivity such as the default mode network was less pronounced. This suggests that meaningful metabolic organization may be most prominent within intermediate frequency ranges (e.g. 0.01–0.1 Hz). In contrast, the 0.001–0.01 Hz band showed a random-like pattern.

**Figure 4.**
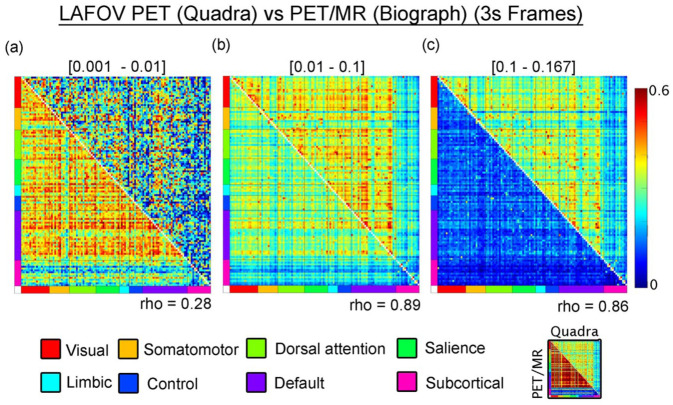
Comparison of metabolic connectivity metrics derived from a conventional PET/MR system (lower triangle) and a next-generation LAFOV PET/CT system (upper triangle) using the CompCor method: (a) connectivity estimates in the lower frequency band (0.001–0.01 Hz) show a weak linear relationship between scanners (Spearman’s rho = 0.28), indicating poor correspondence in this range, (b) in the mid-frequency band (0.01–0.1 Hz), however, a strong linear correlation is observed between the two systems (rho = 0.89), suggesting improved consistency across scanner types, and (c) the higher band (0.1–0.16 Hz) also shows a high correlation between systems (rho = 0.86), although connectivity estimates from the PET/MR system are generally weaker in magnitude. The lower strength of connectivity observed in the PET/MR data aligns with findings from previous studies using standard PET systems (e.g.^11,15^). Connectivity matrices are organized according to large-scale functional networks described by Thomas Yeo et al.,^48^ along with subcortical regions from the Harvard–Oxford atlas.^60^

To examine the impact of scanner sensitivity on M-MC using the CompCor method, we compared results from the next-generation LAFOV PET/CT system with those from a standard Siemens PET/MR hybrid scanner (Figure 4). Both, 0.01–0.1 and 0.1–0.16 Hz bands yielded high inter-scanner correlations (rho = 0.89 and rho = 0.86, respectively). Nonetheless, connectivity strength was consistently greater in data acquired with the next-generation LAFOV PET/CT system, indicating enhanced sensitivity to spontaneous metabolic fluctuations. Generally, the observed connectivity amplitudes are consistent with previously reported connectivity estimates from standard PET systems^11,15^ and underscore the improved sensitivity of the LAFOV PET/CT scanner. In the 0.001–0.01 Hz band, scanner-derived connectivity metrics showed limited correspondence (rho = 0.28). Of note, PET/MR data exhibited the highest connectivity in this band, which however seems to be attributed to the lower sensitivity and lower sampling rate (see discussion).

**Figure 5.**
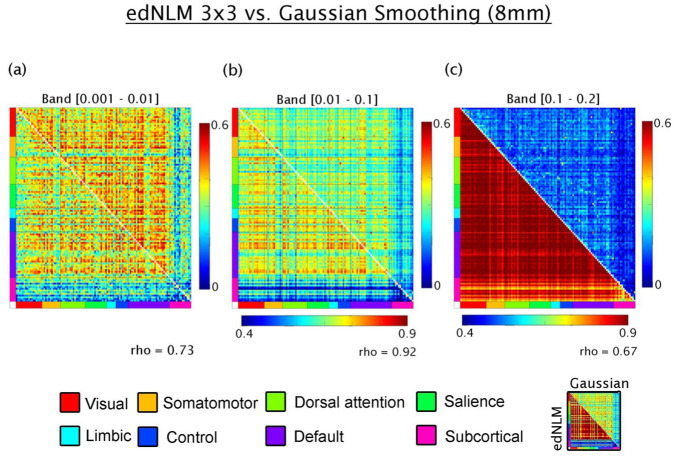
Comparison of filtering techniques applied to high-temporal resolution metabolic connectivity data acquired with a current-generation PET/MRI scanner: (a) in the lowest frequency band (0.001–0.01 Hz), metabolic connectivity estimates processed with edNLM filtering (upper triangle) show comparable magnitude to those processed with conventional Gaussian smoothing (lower triangle), with a moderate linear correlation between methods (Spearman’s rho = 0.72), (b) in the 0.01–0.1 Hz band, edNLM filtering yields noticeably higher connectivity values compared to Gaussian smoothing, although the correspondence between methods remains high (rho = 0.91), and (c) in the higher frequency band (0.1–0.2 Hz), the linear association remains moderate (rho = 0.72), but the edNLM-processed data consistently overestimate connectivity relative to the Gaussian approach. Connectivity values are grouped according to large-scale functional networks defined by Thomas Yeo et al.,^48^ along with subcortical regions from the Harvard–Oxford atlas.^60^ edNLM: extended dynamic non-local means.

**Figure 6.**
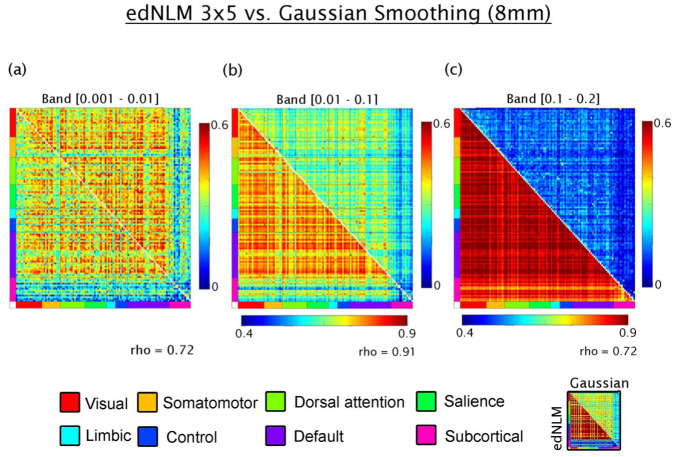
Comparison of signal processing techniques applied to high-temporal resolution metabolic connectivity data acquired with a current-generation PET/MRI scanner: (a) in the lowest frequency band (0.001–0.01 Hz), metabolic connectivity estimates processed with edNLM filtering (upper triangle) show comparable magnitude to those processed with conventional Gaussian smoothing (lower triangle), with a moderate linear correlation between methods (Spearman’s rho = 0.73), (b) in the 0.01–0.1 Hz band, edNLM filtering yields noticeably higher connectivity values compared to Gaussian smoothing, although the correspondence between methods remains high (rho = 0.92), and (c) in the higher frequency band (0.1–0.2 Hz), the linear association remains moderate (rho = 0.67), but the edNLM-processed data consistently overestimate connectivity relative to the Gaussian approach. Connectivity values are grouped according to large-scale functional networks defined by Thomas Yeo et al.,^48^ along with subcortical regions from the Harvard–Oxford atlas.^60^ edNLM: extended dynamic non-local means.

Next, the influence of spatial filtering methods was assessed. In the lowest frequency band (0.001–0.01 Hz), edNLM and Gaussian-smoothed data showed a moderate correlation (rho = 0.72), with comparable connectivity magnitudes. For the 0.01–0.1 Hz band, edNLM filtering yielded highly elevated connectivity values, yet a strong linear relationship was preserved between methods (rho = 0.91). In the highest frequency band (0.1–0.2 Hz), M-MC was again more pronounced in data processed with the edNLM filter. Although the spatial pattern of connectivity remained moderately consistent with Gaussian smoothing (rho = 0.72), the overestimation introduced by edNLM rendered the resulting M-MC values less comparable to those reported in previous literature (see Figure 5). A similar pattern was observed when the temporal window of the edNLM filter was reduced (Figure 6), further indicating that the degree of temporal smoothing applied prior to M-MC estimation exerts a substantial influence on the connectivity strength.

**Figure 7.**
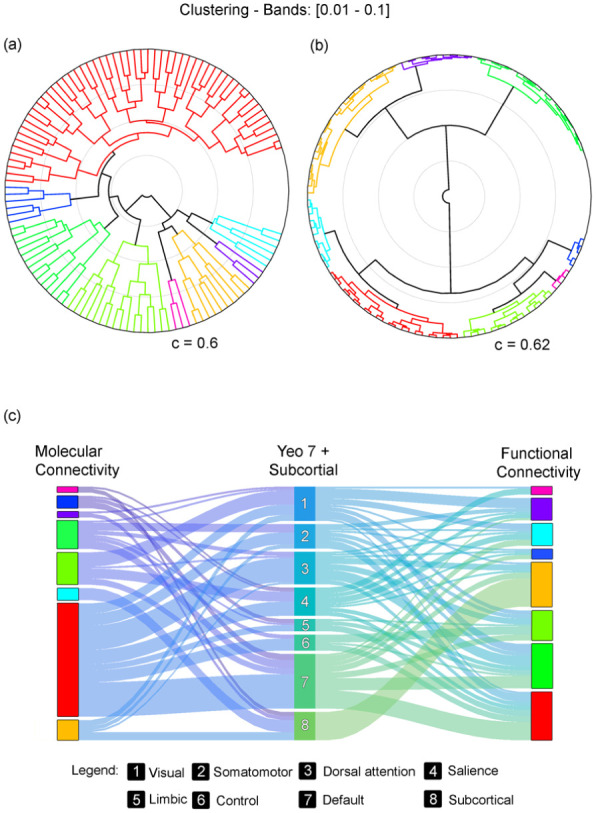
Comparison of metabolic and functional connectivity network clustering: (a) hierarchical clustering of metabolic connectivity data acquired from the next-generation PET/CT scanner reveals distinct network structures, (b) a corresponding dendrogram illustrates the functional connectivity networks derived from fMRI data. While both modalities show clear modular organization, metabolic connectivity clusters are positioned more closely together, suggesting higher inter-network similarity compared to the more differentiated structure observed in functional connectivity. The cophenetic correlation coefficients were similar across modalities, with *c* = 0.60 for metabolic connectivity and *c* = 0.62 for functional connectivity, indicating comparable consistency in the hierarchical cluster solutions, and (c) a Sankey plot visualizes the correspondence between clustered metabolic networks and canonical functional networks as defined by Thomas Yeo et al.^48^ Notably, one prominent metabolic cluster (colored red) spans across all Yeo-defined networks, suggesting a degree of global integration in the metabolic architecture not observed in the functional domain.

Hierarchical clustering revealed differing organizational properties of metabolic and hemodynamic connectivity networks (Figure 7). Dendrograms constructed from fPET and fMRI connectivity matrices showed that while both modalities exhibited distinct network boundaries, M-MC clusters appeared more spatially cohesive and less dispersed. The cophenetic correlation coefficients of the resulting hierarchical trees were comparable (fPET: *c* = 0.60; fMRI: *c* = 0.62), indicating similar internal consistency.

To facilitate direct comparison, both datasets were further partitioned into eight clusters using agglomerative clustering, reflecting seven canonical Yeo networks and one subcortical cluster. A Sankey diagram revealed partial overlap between the metabolic and functional clusters (Figure 7(c)), with the largest metabolic cluster spanning across all major Yeo networks, suggesting broader cross-network metabolic integration relative to fMRI.

The mean reliability across permutations for the CompCor method varied across frequency bands. For the 0.001–0.01 Hz band, the mean reliability was *r* = 0.104, with a 95% confidence interval (CI) of 0.082–0.123. For the 0.01–0.1 Hz band, the mean reliability was *r* = 0.957 (CI = 0.952–0.962), and for the 0.1–0.2 Hz band, the mean reliability was *r* = 0.957 (CI = 0.952–0.961). For the band-pass filtering method, the mean reliability was *r* = 0.910 for the 0.001–0.01 Hz band (CI = 0.904–0.916). In the 0.01–0.1 Hz band, the mean reliability increased to *r* = 0.935 (CI = 0.938–0.946). For the 0.1–0.2 Hz band, the mean reliability was *r* = 0.929 (CI = 0.911–0.936).

## Discussion

This study provides a robust basis for the investigation of individual-level metabolic connectivity using high-temporal resolution [^18^F]FDG–fPET data. We systematically investigated how scanner sensitivity (next-generation LAFOV PET/CT vs conventional PET/MR), temporal resolution (1 vs 3 s), signal processing approaches (CompCor vs standard bandpass filtering), and spatio-temporal smoothing methods (Gaussian vs edNLM) affect the estimation of M-MC. Our results highlight key insights: (i) M-MC estimates are sensitive to both scanner performance and temporal preprocessing; (ii) the 0.01–0.1 Hz frequency band includes meaningful network patterns, that is, spatially coherent and reproducible connectivity structures that are not dominated by global or non-neuronal signal components^49^ for both scanner systems; (iii) higher sensitivity of next-generation PET systems captures higher-frequency connectivity patterns that are aliased or lost in conventional PET scanners; and (iv) applying advanced smoothing filters (e.g. edNLM) increases signal amplitude but raises concerns regarding comparability with existing literature. Despite the differences in acquisition and preprocessing, we found a high correlation between M-MC matrices across scanners in the middle frequency band, although absolute values diverged systematically.

A central methodological question addressed in this study concerns the preprocessing strategy used to isolate relevant metabolic fluctuations. Standard bandpass filtering lacks the ability to remove structured noise from physiological or scanner-related sources.^28,50,51^ In contrast, the CompCor method, which was originally developed for fMRI,^28^ offers key advantages for fPET by modeling nuisance signals from white matter and cerebrospinal fluid and regressing them from the data parallel to an optional bandpass frequency filtering. Importantly, this approach also suppresses the irreversible tracer uptake component of the FDG signal, which otherwise dominates the low-frequency spectrum without the need to choose a specific frequency band. Furthermore, our results demonstrate that the CompCor method consistently resulted in lower amplitude but more internally consistent and reproducible M-MC estimates for PET/MR data across all frequency bands when compared to bandpass filtering. However, this suppression likely reflects a more conservative and specific isolation of dynamic fluctuations in glucose metabolism, supporting its suitability for fPET applications, particularly when combined with a temporal resolution of 3 s.

Temporal resolution emerged as a decisive factor in capturing meaningful M-MC. The LAFOV PET/CT system’s 1 s framing enabled robust detection of dynamic fluctuations also in the 0.01–0.2 Hz range. In contrast, PET/MR data acquired at 3 s resolution were inherently limited by a lower Nyquist frequency (~0.167 Hz), resulting in aliasing of higher-frequency components. These aliased signals can misleadingly appear in low-frequency bands, as demonstrated by observed peaks around 0.033 Hz in the PET/MR data (Figure 3(a) vs (c), lower triangles). Theoretical analysis confirmed that high-frequency physiological or vascular signals (e.g. respiratory signals around 0.3 Hz) can alias into this lower range due to undersampling. For a sampling interval of 3 s (PET/MR), the Nyquist frequency is fn=1(2.TR)=1(2.3s)≈0.167Hz . Frequencies above this threshold are folded back into lower bands according to the aliasing formula falias=|f−n.fs|,wherefs=1TR is the sampling rate. As a result, aliased components appear within the 0.001–0.01 Hz range and contaminate the lower bands.^52^ This presents an interpretability issue, as aliased frequencies may mimic low-frequency neural connectivity patterns, including those traditionally attributed to resting-state networks. Consequently, accurate characterization of M-MC, especially in higher frequency bands, requires high temporal resolution to avoid spectral distortion.

The reliability analysis provides important context for interpreting differences between preprocessing strategies. While both CompCor and band-pass filtering yielded high within-scan reliability in the both intermediate and higher frequency ranges, CompCor’s reliability was markedly reduced in the lowest band. In contrast, band-pass filtering produced relatively high reliability even in this frequency band, where network structure and inter-scanner correspondence were weak. This suggests that high reliability alone does not imply physiological specificity, and that stable but non-informative signal components can dominate uncorrected low-frequency fPET data. Beyond its favorable reliability profile, a practical advantage of CompCor is that it does not require a priori selection of band-pass parameters. This is particularly relevant for high-temporal-resolution fPET, where the frequency range of biologically meaningful metabolic fluctuations is not yet well established and may vary across scanners and protocols. By removing structured non-neuronal variance and uptake-related trends without imposing fixed frequency cut-offs, CompCor provides a parsimonious and assumption-light framework for metabolic connectivity analyses.

While the application of temporally-aware smoothing filters (e.g. edNLM) have been shown to improve fPET outcome parameters for task-specific analyses,^15^ our findings caution against the use of temporal smoothing methods in high-temporal M-MC research. Although edNLM-filtered data showed strong correlations with Gaussian-smoothed data across bands (e.g. rho = 0.72–0.91), the absolute values of M-MC became significantly inflated, particularly in the higher bands (0.1–0.2 Hz). This inflation renders direct comparisons with previously published PET connectivity metrics difficult and raises questions about the biological interpretability of such enhanced signals. Similar effects were seen when reducing the temporal component of the edNLM filter, underscoring the strong, potentially detrimental, influence of temporal smoothing on connectivity outcomes. Thus, while advanced filters may be valuable for enhancing spatial consistency, they must be calibrated carefully to preserve physiological interpretability and cross-study comparability in M-MC. We note that alternative filtering strategies beyond those evaluated here have been proposed to improve signal-to-noise characteristics in dynamic PET data, including data-driven and model-based approaches that explicitly account for tracer kinetics and noise structure.^53^

Interestingly, M-MC components, particularly those within the 0.01–0.1 Hz band, were still accurately detectable in PET/MR data despite the lower frame rate and scanner sensitivity.^11,15^ This reflects the robustness of certain connectivity patterns. However, the lower sampling rate (3 s) and sensitivity made further spectral decomposition of other frequency bands unreliable. This finding underscores a subtle yet important point that while the spatial patterns of M-MC may persist at lower temporal resolutions, accurate frequency-resolved analysis depends critically on adequate sampling.

Furthermore, discrepancies in connectivity observed in the 0.001–0.01 Hz band in PET/MR data may be attributed to aliasing of higher-frequency signals. As previously discussed, respiratory or vascular dynamics in the 0.2–0.4 Hz range,^54^ well above the Nyquist threshold of PET/MR data, may alias into the 0.001–0.01 Hz range, falsely suggesting low-frequency connectivity. The data acquired using the next-generation scanner, with its higher frame rate of 1 s, was able to avoid such artifacts and allowed these signals to be more accurately assigned to their true spectral domain. Thus, spectral peaks <0.01 Hz in the PET/MR data should be interpreted cautiously, as they may represent aliased higher-frequency physiological processes rather than genuine resting-state metabolic activity.

A notable observation was that connectivity matrices derived from fPET and fMRI showed a similar modularity, both resolving canonical functional networks, but differed markedly in spatial organization. Specifically, M-MC clusters derived from fPET were more spatially cohesive and less dispersed than those from fMRI. This pattern likely reflects fundamental differences in the underlying signals: while fMRI captures rapid hemodynamic fluctuations linked to neuronal activity, fPET reflects fluctuations in regional glucose metabolism, which may be governed by integrative processes such as synaptic maintenance, glial-neuronal interactions, or neuromodulatory tone.^24,55–57^ This observation supports earlier findings that removing non-neuronal signals enhances the spatial cohesion of metabolic connectivity networks, producing more compact metabolic clusters.^20^ We speculate that these ubiquitous metabolic processes may result in broader and more spatially homogeneous connectivity patterns, however, the actual underlying mechanisms still need to be assessed in future work. Nevertheless, the cophenetic correlation coefficients for both modalities were comparable (*c* ~0.6), suggesting that both hierarchies are internally consistent, albeit representing different neurobiological processes.

Several limitations warrant consideration. First, although our results emphasize the superior temporal resolution of the LAFOV scanner, these benefits are only realized when combined with appropriate preprocessing and filtering strategies. Second, while we used CompCor and bandpass filtering as two representative approaches, the space of denoising methods for fPET is expanding and may benefit from hybrid or adaptive techniques in the future. Third, absolute connectivity values depend on normalization, smoothing, and segmentation procedures, which may vary. Lastly, the interpretation of M-MC, while increasingly supported by emerging literature,^10,24^ remains less established than in fMRI, necessitating further work to identify the underlying neurophysiological processes. Partial volume correction (PVC) was not applied because it mainly affects time-invariant spatial scaling, so leaving it out should not substantially bias correlation-based connectivity estimates. Applying PVC to dynamic PET can also amplify noise and create instability, especially at high temporal resolution. Therefore, PVC was omitted to preserve temporal signal fidelity, in line with prior fPET connectivity studies.^11^ Nevertheless, our work demonstrates that metabolic connectivity from high-temporal resolution fPET data can be reliably estimated from ultra-high-sensitivity LAFOV and conventional scanner systems.

In sum, we provide a comprehensive comparison of M-MC estimation across PET scanner generations and preprocessing pipelines. Our findings demonstrate that ultra-high sensitivity PET systems with high temporal resolution yields high stability of M-MC estimation across random data splits, particularly in higher frequency bands. However, methodological choices particularly regarding filtering and denoising profoundly influence the amplitude, spatial organization, and interpretability of connectivity metrics. Our results support the use of high-temporal-resolution fPET, combined with physiologically-informed preprocessing to enhance the reliability and comparability of M-MC estimates. Altogether, this opens a new avenue in molecular imaging, enabling the assessment of metabolic connectivity at rest and during cognitive, interventional or pharmacological stimulation as well as the re-organization of metabolic network interactions in various brain disorders.
